# Supplementary material for: Association between oral microbiome and seven types of cancers in East Asian population: a two-sample Mendelian randomization analysis
Source: Front Mol Biosci. 2023 Nov 21;10:1327893. doi: 10.3389/fmolb.2023.1327893 (PMC10702768; doi:10.3389/fmolb.2023.1327893)
Supplement: Supplementary file 1 [file DataSheet1.ZIP › Supplementary Materials/MR plots of saliva/saliva═╝/Gastric cancer/pheno.1154_to_gastric cancer_forest.pdf]

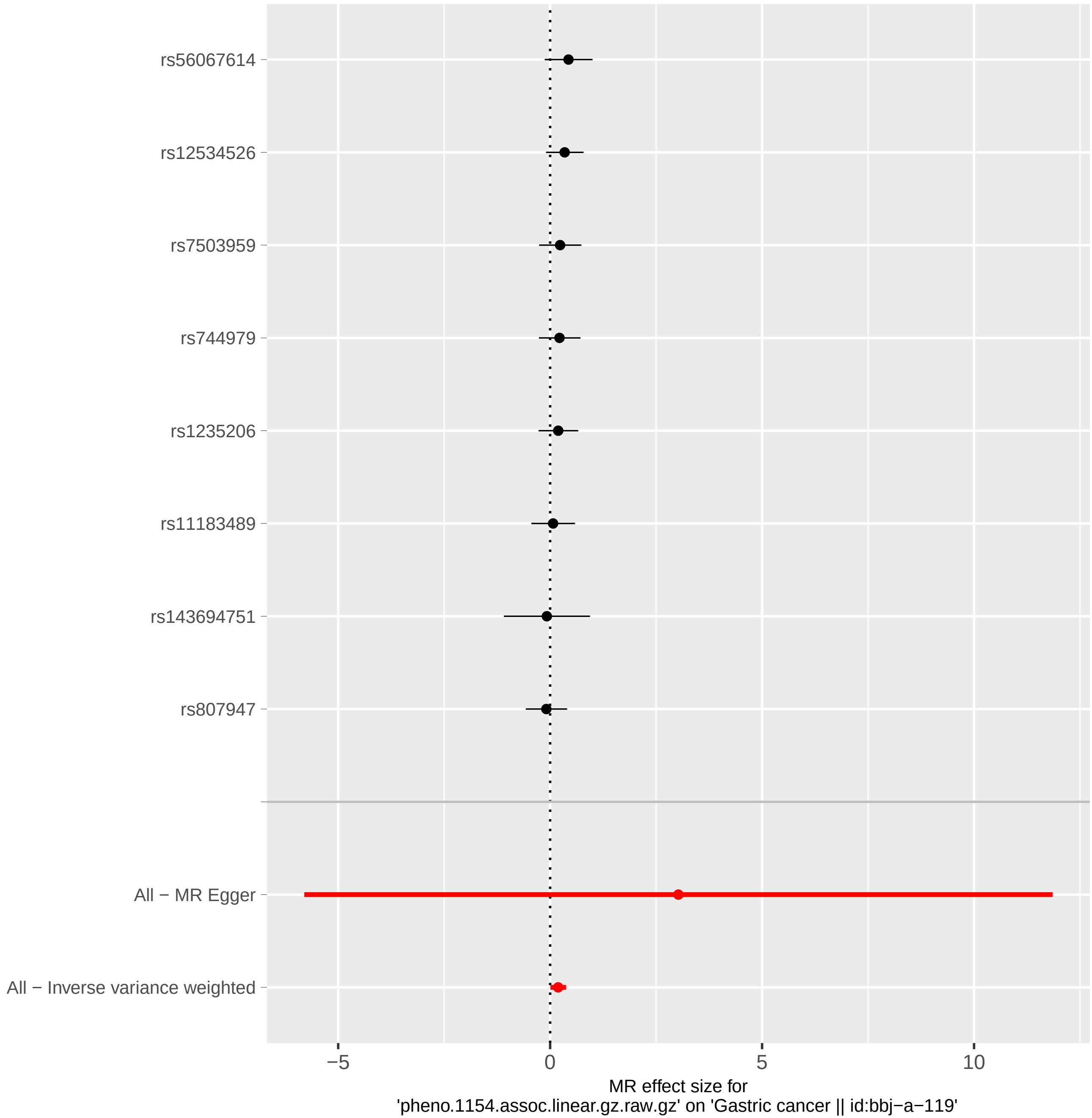
rs56067614
rs12534526
rs7503959
rs744979
rs1235206
rs11183489
rs143694751
rs807947
All − MR Egger
All − Inverse variance weighted
−5
0
5
10
MR effect size for
'pheno.1154.assoc.linear.gz.raw.gz' on 'Gastric cancer || id:bbj−a−119'
